# Exploring the potential of foliar phytohormone application for mitigating water deficit in wheat cropping systems under arid soils

**DOI:** 10.1371/journal.pone.0342914

**Published:** 2026-02-24

**Authors:** Ahmed A. Abdelrhman, Mohamed E. Fadl, Nazih Y. Rebouh, Mohamed Hefzy, Mostafa A. S. AbdElgalil

**Affiliations:** 1 Department of Soils and Water, Faculty of Agriculture, Al-Azhar University, Assiut, Egypt; 2 Division of Scientific Training and Continuous Studies, National Authority for Remote Sensing and Space Sciences (NARSS), Cairo, Egypt; 3 Department of Environmental Management, Institute of Environmental Engineering, RUDN University, Moscow, Russia; 4 Water Requirement and Field Irrigation Research Department, Soils, Water and Environment Research Institute, Agricultural Research Center, Giza, Egypt; 5 Central Laboratory of Organic Agriculture, Agricultural Research Center, Giza, Egypt; Graphic Era Institute of Technology: Graphic Era Deemed to be University, INDIA

## Abstract

In arid regions, water scarcity prompts the overuse of chemical growth regulators, posing ecological and health risks. This study investigates foliar application of microbial phytohormones as a sustainable alternative to mitigate the water deficit’s negative impact on wheat growth and enhance crop productivity. Field experiments over two seasons evaluated the impact of microbial phytohormones microbial gibberellic acid (MGA3) and microbial ascorbic acid (MASA) on wheat yield and water productivity under three deficit irrigation levels: 100%, 80%, and 60% of crop evapotranspiration (ETc). The treatments significantly influenced growth, grain yield, and associated characteristics. Deficit irrigation adversely affected wheat growth and yield. However, MGA3 and MASA foliar treatments significantly improved plant height, flag leaf area and yield components under water stress. The highest grain yield (4.27 t ha ⁻ ¹) was achieved with 100% ETc + MGA3, while the highest irrigation water productivity (IWP, 1.16 kg m ⁻ ³) was recorded with 60% ETc + MGA3. Redundancy analysis confirmed MGA3’s superiority over MASA and control (CK) in enhancing grain yield and crop water productivity (CWP), which were strongly correlated with biological yield and seed index. This study concludes that microbial phytohormones, particularly MGA3, are effective agronomic tools for sustaining wheat productivity in water-scarce arid environments.

## Introduction

Wheat (*Triticum aestivum* L.) is a cornerstone of food security in Egypt and a significant cereal plant within the *Poaceae* family, where it accounts for over 35% of the daily caloric intake. However, domestic production meets only 50% of the annual demand (20 million tons), necessitating costly imports [1]. The Food and Agriculture Organization (FAO) [2] indicates that wheat is the most widely grown crop by area in Egypt. However, local wheat production is inadequate to satisfy the increasing yearly demand, resulting in a disparity between supply and consumption [3]. This shortfall is exacerbated by severe water scarcity, as agriculture consuming 80% of Egypt’s Nile-derived freshwater amidst declining allocations. Furthermore, approximately 60% of the country’s arable land consists of arid soils with low water retention [2]. This shortfall can be addressed by expanding the sown area and improving yield per unit. To expand wheat cultivation, most newly reclaimed soils in Egypt are sandy or sandy calcareous, with 23–30% classified as calcareous [4], indicating a notable drop in productivity [5]. Climate change is projected to intensify these constraints [1,6–8], resulting in decreased water availability for agriculture in arid and semi-arid areas such as Egypt [9]. At present, water scarcity significantly hampers the expansion of wheat cultivation in Egypt [10].

Drought stress significantly impairs wheat physiology and productivity, threatening sustainable food production [11,12], especially in arid regions [13]. Inadequate water stress hinders the availability of vital nutrients and negatively impacts plant development by creating water scarcity [14]. Studies show that different wheat cultivars vary in terms of seed production, harvest index, and plant height, with a significant decline noted in drought-stressed conditions [15,16]. The rising freshwater shortages and worsening droughts from climate change have prompted research into water-efficient irrigation strategies to maximize ‘crop per drop’ [17,18]. Therefore, evaluating practical and environmentally acceptable methods to mitigate the negative effects of water deficit stress on wheat is essential in arid conditions under climate change [19,20]. An effective approach to achieve this is to reduce water loss through transpiration, which may be enhanced by the use of plant hormones. This study tries to identify and produce efficient solutions for optimizing water consumption in agriculture and enhancing food safety in Egypt.

Some studies have indeed demonstrated the potential of deficit irrigation in the region. For example, Saad et al., 2023 [15] demonstrated the feasibility of deficit irrigation in Egyptian wheat systems, achieving yield stability under 20–30% reduced irrigation, However, these studies also highlight critical limitations of deficit irrigation in Egypt; the significant yield penalty (typically 10–30%) under severe water restriction, increased soil salinity risks in arid zones due to reduced leaching, and variable performance across different soil types.

Efficient irrigation management can enhance agricultural production in water-scarce areas, assisting in mitigating issues in these susceptible locations. Deficit irrigation (DI) maximizes water efficiency and reduces water use, enabling crops to endure moderate water stress with minimal impact on production and quality [21,22]. Indeed, studies in Egypt have demonstrated the potential of DI. For instance, Saad et al., 2023 [15] reported that applying 70–80% of crop ETc could maintain stable wheat yields in arid conditions. However, their study also highlighted a critical limitation: more severe water deficits (e.g., 50% ETc) led to significant yield penalties. This underscores the need for complementary agronomic practices to mitigate the negative impacts of stricter water-saving regimes. The application of foliar phytohormones presents one such promising strategy to bolster plant resilience under deficit irrigation. Foliar phytohormone application has proven to be one of the most effective strategies for optimizing water use and enhancing water productivity [23,24]. On the other hand, many studies have noted that water stress negatively affects plant metabolism, significantly reducing crop productivity [15]. Thus, effective management of irrigation water use is crucial for improving water productivity and achieving economic benefits in the water-food nexus [25]. Enhancing CWP and maintaining good yields in arid regions are crucial to ensure food security [26–28].

Irrigation scheduling based on actual ETc is a promising crop water management technique that remains insufficiently investigated in arid areas [29]. The accuracy of the ETc estimate depends on the approach and quality of meteorological data. Although ETc is essential for comprehending energy and water relationships, direct assessment is difficult. Techniques to determine ETc include Eddy-Covariance, lysimeters, water balance, energy balance, Penman-Monteith, and hydrological models [30].

The application of phytohormones presents a promising strategy to mitigate drought stress [14,31]. Phytohormones such as gibberellic acid (GA3) and ascorbic acid (ASA) can regulate stomatal closure, water balance, and antioxidant defense systems, thereby enhancing crop growth and production under water stress [5,32–36]. GA3 and ASA are globally recognized for their ability to stimulate plant growth and serve as protection under stress conditions [37,38] GA3 is a natural plant hormone, a tetracyclic dihydroxy-γ-lactone with the formula C_19_H_22_O_6_. ASA is a successful antioxidant that protects plants from oxidative damage induced by reactive oxygen species produced under drought stress [32]. GA3 increases the rate of seed germination under stress conditions, encourages root growth, and improves water and mineral uptake [39]. Additionally, it controls cell elongation and division, enhances chloroplast development, inhibits the breakdown of chlorophyll, and reduces reactive oxygen species, which are responsible for cell death [40,41]. Foliar treatment of ASA mitigates water stress impacts, enhancing stomatal function, nutrient uptake, plant growth, and productivity under water stress [37,42]. Barahouei et al., 2025 [11] reported that the use of ASA on maize enhanced its physiological characteristics and yield.

Given escalating population growth and demands, the intensifying water scarcity, and the declining crop productivity, it is imperative to enhance water use efficiency across all irrigation methods and soil types, particularly coarse-textured soils characterized by high infiltration rates and low water retention capacity, to optimize our water resources. Furthermore, it is important to maintain and improve crop yield under water constraint by employing natural plant growth enhancements that include beneficial and safe attributes to maintain soil sustainability and ecological security [43,44]. Phytohormones such as GA3 and ASA can regulate stomatal closure, water balance, and antioxidant defense systems, thereby enhancing crop growth and production under water stress.

While synthetic phytohormones are widely used, their production can be energy-intensive and environmentally costly. Microbial synthesis of phytohormones offers a sustainable alternative, leveraging the metabolic capabilities of fungi and yeast to produce biologically active compounds. Microbial-derived phytohormones may also contain complementary bioactive metabolites (e.g., polysaccharides, enzymes, siderophores) that can enhance plant stress resilience through synergistic effects. In this study, we utilized microbially produced GA3 and ASA from *Fusarium incarnatum* and *Saccharomyces cerevisiae*, respectively, to evaluate their efficacy in improving wheat drought tolerance under deficit irrigation in arid calcareous soils [32].

Therefore, this study hypothesized that foliar application of microbially-sourced phytohormones (GA3 and ASA) would significantly enhance the drought tolerance of wheat, leading to improved growth, yield, and water-use efficiency under deficit irrigation in arid soils. Accordingly, this study aims to 1) quantify the effects of foliar-applied microbial GA3 (MGA3) and ASA (MASA) on wheat growth, yield, and irrigation water use efficiency under different irrigation levels (100%, 80%, and 60% ETc), 2) determine the potential of these phytohormone to enable more severe water-saving strategies without significant yield loss, thereby addressing a critical gap in sustainable agriculture for water-scarce regions.

## Materials and methods

### Experimental site and design

**Fig 1:**
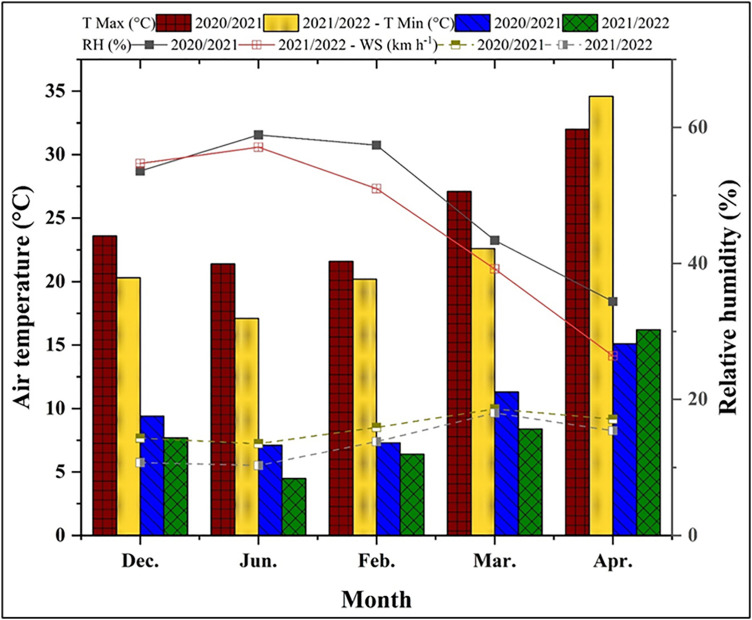
Average monthly meteorological data from Assiut weather station during the two growth winter seasons of 2020/21 and 2021/22; maximum and minimum air temperature (bars); Monthly relative humidity (RH) and wind speed (WS), (line).

Field experiments were conducted over the 2020/21 and 2021/22 winter seasons at the Arab Al-Awammer Research Station, Assiut, Egypt. The mean monthly climatic data for experimental sites during the two growing seasons of 2020/2021 and 2021/2022 are presented in Fig 1. According to Soil Taxonomy (Soil Survey Satff, 2022) [45], the soil is classified as sandy calcareous (*Typic Torripsamments*); its physical and chemical properties are summarized in Table 1 as determined by recognized methods from Jackson, 1973 [46] and Sparks et. al., 2020 [47]. A drip irrigation system was established using 16 mm diameter GR polyethylene pipe, featuring auto-emitters every 30 cm along the pipe and 50 cm between laterals, with a flow rate of 4 liters per hour (L h ⁻ ¹) at 1.5 bar.

**Table 1:** Physical and chemical properties for the soil experiment.

Property		Unit	Mean value
Gravel		%	32
Sand		%	**91.20**
Silt		%	5.30
Clay		%	3.50
Texture Class			**Sandy**
Saturation percentage		%	23.5
Field capacity		%	10.8
Wilting point		%	4.60
Available water		%	6.70
Bulk density		Mg cm^-3^	1.55
CaCO_3_		%	35.18
Organic Matter		%	0.41
pH (1−1)			8.45
EC (Soil past extract)		ds m^-1^	0.48
Soluble cations	Ca	mmol L^-1^	1.58
	Mg	mmol L^-1^	1.24
	Na	mmol L^-1^	0.37
	K	mmol L^-1^	0.77
Soluble anions	CO_3_+ HCO_3_	mmol L^-1^	1.87
	Cl	mmol L^-1^	1.62
	SO_4_	mmol L^-1^	0.39
Available N		mg kg^-1^	31.35
Available P		mg kg^-1^	7.81
Available K		mg kg^-1^	39.86

**Fig 2:**
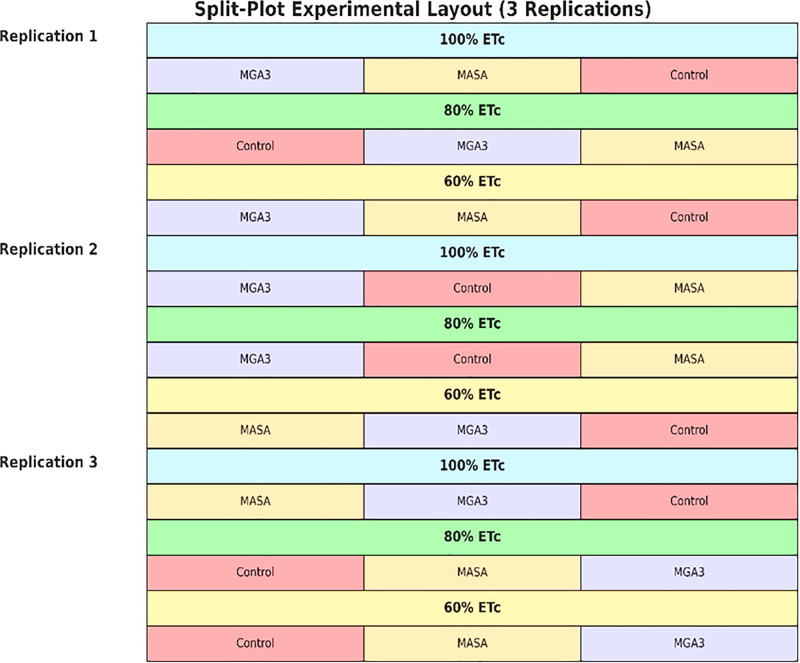
A schematic diagram of a split-plot design.

A split-plot design with three replications was employed (Fig 2). Irrigation regimes (100%, 80%, and 60% of ETc) were randomly assigned to the main plots, and the foliar spray treatments (CK, MGA3, and MASA) were randomly assigned to the sub-plots within each main plot. This resulted in a total of 27 experimental plots (3 irrigation levels × 3 phytohormone treatments × 3 replications).

Each sub-plot consisted of eight ridges, each 3 meters long and spaced 50 cm apart, resulting in a total sub-plot area of 12 m² (8 ridges × 3 m × 0.5 m). A buffer zone of 1 meter was maintained between main plots and 0.5 meters between sub-plots to minimize edge effects and cross-irrigation. The wheat cultivar ‘Masr-1’ was sown on the ridges at a seeding rate of 190 kg ha ⁻ ¹, with seeds hand-sown along the furrows to achieve a uniform plant stand. The total area of the experimental field, including all plots and buffers, was approximately 500 m².

### Agronomic practices

The Wheat cultivar (Masr-1) was sown in early December each season at a rate of 190 kg/ha. Wheat plants were harvested on April 27 and May 3 in the first and second seasons, respectively. Standard agricultural practices were followed as per the recommendations of the Egyptian Ministry of Agriculture. Nitrogen, phosphorus, and potassium were applied at 220, 75, and 120 kg ha^-1^, respectively, utilizing urea (46% N), triple superphosphate (46% P_2_O_5_), and potassium sulfate (50% K_2_O) as nutrient sources. Treatments were applied using the foliar spray method. The treatment solution was applied at a rate of 480 L ha^-1^ with two sprays: the first 30 days after planting and the second 20 days later (50 days post-Planting). All treatments included the wetting agent Triton B, and a knapsack hand sprayer was applied to the wheat plants.

### Crop evapotranspiration (ETc) and irrigation scheduling

Reference evapotranspiration (ET₀) was calculated using the FAO Penman-Monteith method [48]. with daily meteorological data (solar radiation, air temperature, humidity, and wind speed) obtained from an on-site weather station. The CROPWAT 8.0 model was used for these calculations.

The crop evapotranspiration (ETc, mm day ⁻ ¹) was then determined for wheat at different growth stages using Equation 1 [49].


$$ET_{c}\;=\;ET_{\text{0}}\;\times \;Kc$$ (1)

where; Kc, Crop coefficient. The Kc values applied were: 0.7 (initial stage), 1.15 (mid-season stage), and 0.4 (late-season stage).

### Actual irrigation water applied (AIWA)

The total AIWA (mm, per irrigation interval) for each treatment was calculated based on ETc, a 10% leaching fraction (Lf), and irrigation efficiency of the drip system (Er), estimated at 90% based on manufacturer specifications and system maintenance records [50] using the following Equation 2:


$$AIWA\;=\;\frac{ETc+Lf}{Er}$$ (2)

The total seasonal irrigation water applied for each treatment across both growing seasons is presented in Table 2. Irrigation was applied when the cumulative ETc reached 30 mm, ensuring that the soil moisture deficit in the root zone was replenished according to the designated treatment level (100%, 80%, or 60% of the calculated volume).

**Table 2:** Total seasonal irrigation water applied (mm) for each treatment during the 2020/2021 and 2021/2022 growing seasons.

Treatment	2020/2021 Season	2021/2022 Season
100% ETC	555.4	506.6
80% ETC	444.3	405.3
60% ETC	333.3	304.0

The IWP and CWP were calculated as the ratio of grain yield to AIWA and ETc, respectively (Equations 3 and 4) [51]:


$$\text{IWP}=\frac{\text{Total grain yield }(kg\;{ha}^{-\text{1}})}{\text{Irrigation Water Applied }({\text{m}}^{\text{3}}\;{ha}^{-\text{1}})}$$ (3)

$$\text{CWP}=\frac{\text{Total grain yield }(\text{kg }{ha}^{-\text{1}})}{\text{Crop evapotranspiration }({\text{m}}^{\text{3}}\;{ha}^{-\text{1}})}$$ (4)

### Microbial phytohormones preparations and characterization

#### Microbial gibberellic acid (MGA3) production and quantification.

Gibberellic acid was produced by the endophytic fungus *Fusarium incarnatum* ASU19, which was previously isolated from onion roots and identified using ITS sequencing (NCBI accession number MK387876), selected for its known phytohormone-producing capabilities and previous isolation from stressed plant hosts, suggesting adaptive ecological relevance. The use of a microbial source aligns with sustainable agricultural practices by reducing reliance on chemical synthesis. For GA3 production, the fungus was cultivated in a mineral broth containing (g L ⁻ ¹): yeast extract (1.0), glucose (20.0), KH₂PO₄ (5.0), NaNO₃ (2.0), KCl (0.5), and MgSO₄·7H₂O (0.5) [38]. The broth was inoculated with a 2% (v/v) conidial suspension (10⁶ conidia mL ⁻ ¹) and incubated at 28 ± 1 °C for 7 days under continuous shaking (120 rpm). After incubation, the fungal broth was centrifuged at 8,000 × g for 20 min. The supernatant was acidified to pH 2.5 using 1N HCl and extracted twice with an equal volume of ethyl acetate. The combined organic phases were evaporated under reduced pressure at 40°C. The crude GA3 extract was re-dissolved in sterile distilled water and filtered through a 0.22 µm membrane. The GA3 concentration was quantified spectrophotometrically at 254 nm using a standard curve of pure GA3 (Sigma-Aldrich, ≥ 90% purity) ranging from 10 to 100 µg mL ⁻ ¹ (R² = 0.998) [52]. The final concentration was adjusted to 150 mg L ⁻ ¹ for foliar application, a concentration previously demonstrated to be effective in enhancing plant growth under stress conditions in preliminary dose-response trials and supported by literature [38,52].

#### Microbial ascorbic acid (MASA) production and quantification.

Ascorbic acid was produced using *Saccharomyces cerevisiae* ASU211, a yeast strain recognized for its safety, scalability, and potential to produce antioxidants under fermentation conditions. Microbial ASA production offers a renewable and potentially cost-effective alternative to chemical extraction. The yeast was pre-cultured in Yeast Malt Extract (YM) broth for 24 h at 30 ± 1 °C. Cells were harvested by centrifugation (6,000 × g, 15 min), washed, and re-suspended in saline to achieve a concentration of 10⁵ CFU mL ⁻ ¹. For ASA production, a modified medium was used, composed of (g L ⁻ ¹): yeast extract (5.0), peptone (5.0), glucose (20.0), galactose (3.0), and MgSO₄·7H₂O (0.5), with an initial pH of 5.0 [38]. The production medium was inoculated with 2% (v/v) of the yeast suspension and incubated at 30 ± 1 °C for 72 h under shaking (150 rpm). The broth was then centrifuged at 6,000 × g for 15 min at 4 °C. ASA in the supernatant was quantified using the 2,6-dichloroindophenol titrimetric method. Briefly, a standard curve was prepared using L-ASA (Sigma-Aldrich, ≥ 99% purity) in the range of 5–50 µg mL ⁻ ¹ (R² = 0.995). The concentration in the supernatant was determined by measuring the absorbance decrease at 515 nm. The final working solution for foliar application was adjusted to 150 mg L ⁻ ¹, a concentration selected based on its proven efficacy in mitigating water stress in wheat as reported in previous studies and confirmed in our initial trials [53].

The selection of microbial sources for phytohormone production was based on their environmental compatibility, potential for on-farm or industrial fermentation, and alignment with circular bioeconomy principles. Using microbially derived phytohormones may also reduce the ecological footprint compared to chemically synthesized analogs.

### Data collection and statistical analysis

Data on plant height, flag leaf area, spike length, number of spikes per m ⁻ ², yield components, and final grain and biological yield were collected. Data from the two growing seasons were analyzed together using a combined split-plot design over environments (seasons), where the ‘season’ was considered a random effect to account for inter-annual variability. The statistical model was presented in the following Equation (5) [54]:


$$Y_{ijkl}=\mu +\alpha_{i}+\beta_{j}+{\left(\alpha \beta \right)}_{ij}+\gamma_{k}+{\left(\alpha \gamma \right)}_{jk}+{\beta \gamma }_{jk}+{\left(\alpha \beta \gamma \right)}_{ijk}+\varepsilon_{ijkl}$$ (5)

where: *Y*_*ijkl*_ is the observed value, μ is the overall mean, α_i_ is the fixed effect of the 1^th^ irrigation level (main plot), β_j_ is the fixed effect of the 2^nd^ season, (αβ)_ij_ is the interaction between irrigation and season, γ_k_ is the fixed effect of the 1^th^ phytohormone treatment (sub-plot), (αγ)_ik_, (βγ)_jk_, and (αβγ)_ijk_ are the two-way and three-way interactions, ε_ijkl_ is the random error associated with the l^th^ replication within the all treatment combination.

Prior to analysis of variance (ANOVA), data were tested for normality (Shapiro-Wilk test, p > 0.05) and homogeneity of variances (Levene’s test, p > 0.05). A three-way ANOVA was performed using the IBM SPSS Statistics package (version 28.0) to evaluate the main effects of irrigation, phytohormone treatment, and season, as well as their interactions. When the ANOVA indicated significant F-values (p < 0.05), treatment means were separated using Tukey’s Honest Significant Difference (HSD) post-hoc test at a 5% significance level. Significant interaction effects between irrigation and phytohormone treatments are reported and interpreted in the results. All data in tables and figures are presented as mean ± standard deviation [55].

## Results

### Weather conditions, evapotranspiration, and irrigation water applied

**Fig 3:**
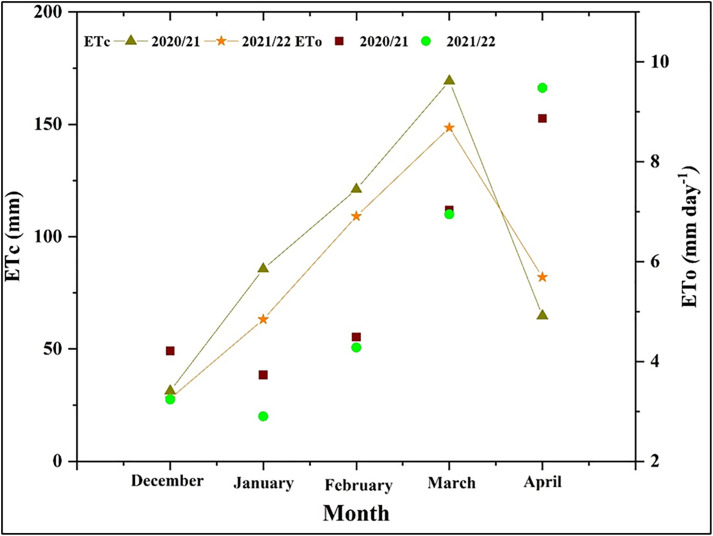
Monthly evapotranspiration (ETo in mm day^-1^) and actual crop evapotranspiration (ETc in mm) for wheat during the 2020/2021 and 2021/2022 seasons.

**Fig 4:**
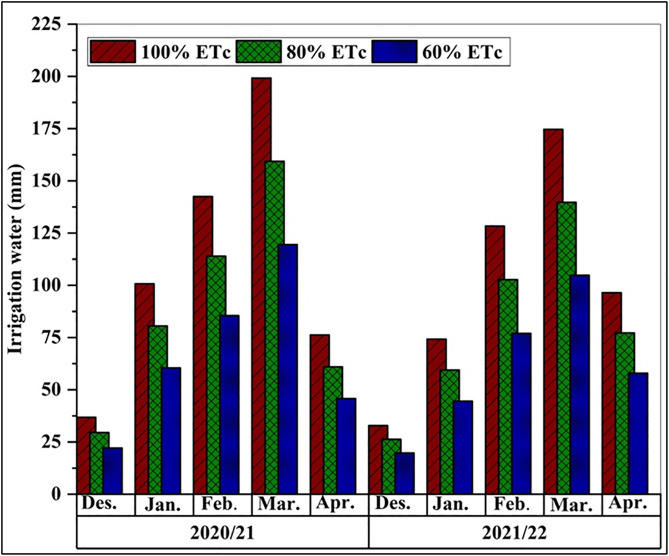
Monthly irrigation water applied (mm) for wheat growth during the 2020/2021 and 2021/2022 seasons. 100% ETc was 100% from crop evapotranspiration; 80% ETc was 80% from crop evapotranspiration; 60% ETc was 60% from crop evapotranspiration.

Climatic conditions differed between the two growing seasons (Fig 1). The 2021/22 season was characterized by lower air temperatures and lower relative humidity during key development phases compared to 2020/21. Consequently, total ETo was higher in the first season (28.33 mm) than in the second (26.85 mm). Crop ETc followed a similar trend, peaking in March and declining towards harvest, with a higher cumulative ETc in the first season (472.11 mm) than the second (430.63 mm) (Fig 3). As designed, the AIWA decreased progressively with increasing irrigation deficit. During the first growing season, AIWA values were 555.42, 444.34, and 333.25 mm and were 506.63, 405.30, and 303.98 mm during the second season, for 100%, 80%, and 60% ETc, respectively. The first season’s AIWA was found to be higher than the second’s (Fig 4).

### Negative effects of deficit irrigation and positive effects of phytohormones on agronomic traits

Deficit irrigation significantly impaired wheat growth and development. Plants receiving 80% and 60% of crop ETc showing a substantial drop in key morphological parameters, including plant height, flag leaf area, spike length, and the number of spikes per m² compared to those cultivated at 100% ETc. The foliar application of microbial phytohormones, however, greatly improved all growth parameters across these varying water levels. MGA3 was particularly effective, producing significantly greater plant heights (p < 0.01) than MASA. A consistent hierarchy of treatment efficacy was observed for plant height, with the tallest plants under 100% ETc + MGA3 and the shortest under 60% ETc without phytohormones. This trend was reflected in the extremes, with the shortest plants recorded at 60% ETc without phytohormones in the first season and the tallest at 100% ETc with MGA3 in the second season, which saw an overall increase in plant height across all treatments. The positive effect of phytohormones was also pronounced on other morphological traits. The application of MGA3 and MASA significantly increased the flag leaf area (p < 0.01) compared to deficit irrigation alone. The maximum flag leaf area was achieved with the combination of 80% ETc irrigation and MGA3 in both seasons, which increased the area by 79% and 76% in the first and second seasons, respectively, compared to the 100% ETc CK (Table 3). Similarly, MGA3 and MASA applications notably increased spike length, which was significantly reduced by lower ETc percentages. The 100% ETc + MGA3 treatment increased spike length by 24% and 22% over the CK in the first and second seasons, respectively (Table 3). Both phytohormones also significantly enhanced the number of spikes per m² across various deficit irrigation levels compared to their corresponding untreated CKs.

**Table 3:** The effects of deficit irrigation and microbial phytohormones on wheat (Masr-1) mean plant height, flag leaf area, spike length, and number of spikes per m² during the 2020/2021 and 2021/2022 seasons.

Treatment	2020/2021				2021/2022				
	100% ETc	80% ETc	60% ETc	Mean	100% ETc		80% ETc	60% ETc	Mean
**Plant height (cm)**									
**CK**	64.93 ± 0.12^g^	62.00 ± 1.00^h^	60.77 ± 0.68^h^	62.57 ± 0.45^c^		64.33 ± 0.58^f^	64.10 ± 0.53^f^	62.87 ± 0.60^f^	63.76 ± 0.04^c^
**GA3**	91.97 ± 0.29^a^	88.05 ± 0.16^b^	85.15 ± 0.61^c^	88.39 ± 0.23^a^		94.07 ± 0.68^a^	92.23 ± 0.68^ab^	90.33 ± 0.58^b^	92.21 ± 0.06^a^
**ASA**	83.03 ± 0.44^d^	79.68 ± 1.10^e^	76.93 ± 0.40^f^	79.88 ± 0.39^b^		85.13 ± 0.72^c^	81.78 ± 1.63^d^	79.03 ± 0.25^e^	81.98 ± 0.70 ^b^
**Mean**	79.98 ± 0.28^a^	76.58 ± 0.76^b^	74.28 ± 0.57^c^			81.18 ± 0.66^a^	79.37 ± 0.95^b^	77.41 ± 0.48^c^	
**Flag leaf area** **(cm²)**									
**CK**	13.32 ± 0.67^f^	12.74 ± 0.64^f^	12.74 ± 0.64^f^	12.93 ± 0.02^c^	13.92 ± 0.69^f^		13.34 ± 0.66^f^	13.34 ± 0.66^f^	13.53 ± 0.02^c^
**GA3**	21.48 ± 1.08^b^	23.89 ± 1.20^a^	19.95 ± 1.00^c^	21.77 ± 0.10^a^	22.08 ± 1.09^b^		24.49 ± 1.21^a^	20.55 ± 1.01^c^	22.37 ± 0.10^a^
**ASA**	18.30 ± 0.92^d^	17.90 ± 0.89^d^	16.25 ± 0.81^e^	17.48 ± 0.05^b^	18.90 ± 0.93^d^		18.50 ± 0.91^d^	16.85 ± 0.83^e^	18.08 ± 0.05^b^
**Mean**	17.70 ± 0.89^b^	18.18 ± 0.91^a^	16.31 ± 0.82^c^		18.30 ± 0.90^b^		18.78 ± 0.93a	16.91 ± 0.83^c^	
**Spike length (cm)**									
**CK**	9.87 ± 0.12^e^	9.41 ± 0.15^ef^	9.11 ± 0.15^f^	9.47 ± 0.02^c^	10.91 ± 0.34^e^		10.45 ± 0.40^ef^	10.15 ± 0.37^f^	10.50 ± 0.03^c^
**GA3**	12.27 ± 0.15^a^	11.52 ± 0.13^b^	11.47 ± 0.13^b^	11.75 ± 0.02^a^	13.30 ± 0.26^a^		12.55 ± 0.22^b^	12.55 ± 0.22^b^	12.78 ± 0.03^a^
**ASA**	11.17 ± 0.13^bc^	10.83 ± 0.08 cd	10.67 ± 0.13^d^	10.89 ± 0.03^b^	12.20b ± 0.31^c^		11.87 ± 0.25 cd	11.70 ± 0.22^d^	11.92 ± 0.05^b^
**Mean**	11.10 ± 0.13^a^	10.59 ± 0.12^b^	10.42 ± 0.13^c^		12.14 ± 0.13^a^		11.62 ± 0.29^b^	11.45 ± 0.27^c^	
**Number of spike/m** ^**2**^									
**CK**	308.67 ± 1.15^f^	297.33 ± 4.62^g^	279.67 ± 0.58^h^	295.22 ± 6.35^c^	294.33 ± 4.04^g^		283.33 ± 2.89^h^	240.67 ± 1.15^i^	361.11 ± 8.08^a^
**GA3**	366.00 ± 1.00^a^	350.00 ± 1.73^b^	337.67 ± 0.58c	351.22 ± 3.31^a^	377.67 ± 2.31a		357.33 ± 1.15b	348.33 ± 0.58^c^	331.78 ± 4.04^b^
**ASA**	334.67 ± 0.58 cd	332.33 ± 0.58^d^	321.33 ± 1.15^e^	239.44 ± 2.31^b^	340.67 ± 0.58^d^		334.00 ± 1.73^e^	320.67 ± 1.15^f^	272.78 ± 3.46^c^
**Mean**	336 ± 0.91^a^	326 ± 2.31^b^	312 ± 0.77^c^		337 ± 2.31a		324.89 ± 1.92b	303.22 ± 0.96^c^	

CK: control, GA3; microbial gibberellic acid (MGA3), ASA; microbial ascorbic acid (MASA). Values are expressed as the mean ± standard deviation (n = 3). Statistically significant differences are indicated by different letters according to Tukey’s multiple range tests at P < 0.05.

The detrimental impact of water deficit also extended to yield formation. On average over both seasons, the 60% ETc treatment led to a decrease in spike weight, grain weight per spike, and 1000 grains weight (seed index) by 16.50%, 19.00%, and 24.50%, respectively, compared to 100% ETc (Table 4). In contrast, the yield parameters of the MGA3 and MASA treatments improved significantly (p < 0.01) throughout all deficit irrigation levels. Although deficit irrigation alone did not noticeably alter the number of grains per spike, both MGA3 and MASA treatments significantly increased it under water stress. The highest increases in grain weight per spike and seed index occurred in the 100% ETc + MGA3 treatment, and the efficacy of treatments on the 1000-grain weight followed the same descending hierarchy as for plant height (Table 4).

**Table 4:** The effects of deficit irrigation and microbial phytohormones on wheat (Masr-1) number of grains per spike, spike weight, grin weight per spike, and weight 1000grin (seed index) during the 2020/2021 and 2021/2022 seasons.

Treatment	2020/2021				2021/2022			
	100% ETc	80% ETc	60% ETc	Mean	100% ETc	80% ETc	60% ETc	Mean
**Number of grains per spike**								
**CK**	50.77 ± 4.35^b^	46.21 ± 3.10^b^	48.21 ± 0.96b	48.40 ± 8.41^b^	48.99 ± 0.58^d^	48.99 ± 2.52^d^	51.10 ± 1.57^d^	49.70 ± 4.67^c^
**GA3**	71.44 ± 1.17^a^	55.10 ± 3.03b	52.44 ± 1.83b	59.66 ± 6.03^a^	87.44 ± 1.83^a^	71.10 ± 1.57^b^	58.21 ± 0.77^c^	72.25 ± 4.18^a^
**ASA**	54.21 ± 1.26^b^	51.44 ± 2.01^b^	51.10 ± 6.20^b^	52.25 ± 9.47^b^	68.10 ± 1.57^b^	58.44 ± 6.17^c^	49.99 ± 1.53^d^	58.84 ± 9.27^b^
**Mean**	58.81 ± 2.26^a^	50.92 ± 2.71^b^	50.58 ± 3.00^b^		68.81 ± 1.33^a^	59.51 ± 3.42^b^	53.10 ± 1.29^c^	
**Spike weight (gm)**								
**CK**	2.83 ± 0.058^e^	2.63 ± 0.058^f^	2.30 ± 0.00^g^	2.58 ± 0.115^c^	3.63 ± 0.115^e^	3.43 ± 0.153^f^	3.10 ± 0.100^g^	3.38 ± 0.368^c^
**GA3**	4.14 ± 0.021^a^	3.50 ± 0.031^c^	3.03 ± 0.021^d^	3.56 ± 0.072^a^	4.49 ± 0.080^a^	4.30 ± 0.122^c^	3.38 ± 0.107^d^	4.36 ± 0.309^a^
**ASA**	3.77 ± 0.061^b^	3.43 ± 0.086^c^	2.70 ± 0.090^ef^	3.30 ± 0.237^b^	4.57 ± 0.157^b^	4.23 ± 0.080^c^	3.50 ± 0.055^ef^	4.10 ± 0.292^b^
**Mean**	3.58 ± 0.046^a^	3.19 ± 0.058^b^	2.67 ± 0.037^c^		4.38 ± 0.188^a^	3.99 ± 0.118^b^	3.47 ± 0.087^c^	
**Grin weight per spike (gm)**								
**CK**	1.93 ± 0.00^de^	1.53 ± 0.006^f^	1.51 ± 0.006^f^	1.66 ± 0.012^c^	2.49 ± 0.058^de^	2.10 ± 0.055^f^	2.08 ± 0.055^f^	2.22 ± 0.168^c^
**GA3**	2.86 ± 0.252^a^	2.28 ± b0.006^c^	1.93 ± 0.006^de^	2.36 ± 0.0263^a^	3.43 ± 0.306^a^	2.85 ± 0.055^bc^	2.71 ± 0.055 cd	3.00 ± 0.416^a^
**ASA**	2.57 ± 0.020^b^	2.14 ± c0.006^d^	1.75 ± 0.006^ef^	2.15 ± 0.032^b^	3.13 ± 0.042^b^	2.32 ± 0.055^ef^	2.50 ± 0.055^de^	2.65 ± 0.152^b^
**Mean**	2.45 ± 0.091^a^	1.99 ± 0.006^b^	1.73 ± 0.006^c^		3.02 ± 0.135^a^	2.42 ± 0.055^b^	2.43 ± 0.055^b^	
**Weight 1000 grin (gm)**								
**CK**	36.86 ± 0.55^e^	35.00 ± 0.46^f^	33.12 ± 0.80^g^	34.99 ± 1.81^c^	44.00 ± 0.26^e^	36.20 ± 0.30^f^	30.48 ± 0.43^g^	36.89 ± 0.99^c^
**GA3**	55.71 ± 0.24^a^	49.73 ± 0.21^b^	45.43 ± 0.21^c^	50.29 ± 0.65^a^	56.20 ± 0.61^a^	48.86 ± 0.06^bc^	49.94 ± 0.52^b^	51.66 ± 1.19^a^
**ASA**	44.66 ± 0.21^c^	40.50 ± 0.00^d^	41.56 ± 0.60^d^	42.24 ± 0.20^b^	45.63 ± 1.03^d^	47.53 ± 0.64^c^	44.73 ± 0.59^de^	45.96 ± 2.25^b^
**Mean**	45.74 ± 0.33^a^	41.74 ± 0.22^b^	40.04 ± 0.54^c^		48.61 ± 0.63^a^	44.20 ± 0.33^b^	41.71 ± 0.51^c^	

CK: control, GA3; microbial gibberellic acid (MGA3), ASA; microbial ascorbic acid (MASA). Values are expressed as the mean ± standard deviation (n = 3). Statistically significant differences are indicated by different letters according to Tukey’s multiple range tests at P < 0.05.

**Fig 5:**
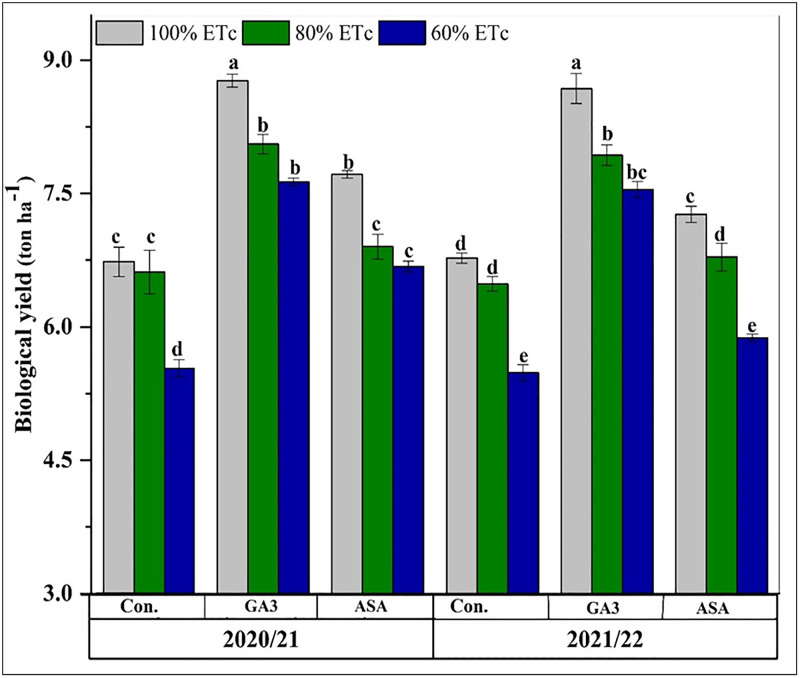
The effects of deficit irrigation and microbial phytohormones on wheat (Masr-1) biological yield during the 2020/2021 and 2021/2022 seasons. CK: control, GA3; microbial gibberellic acid (MGA3), ASA; microbial ascorbic acid (MASA). Data are presented as means, line bars indicate ± standard errors, and the different upper letters demonstrate significant differences at P < 0.05 level according to Tukey’s multiple range tests at P < 0.05.

These improvements in yield components translated directly to higher final output. Reducing irrigation rates notably lowered biological yield in both seasons, with the 60% ETc treatment causing reductions of 18% and 19% in the first and second seasons, respectively (Fig 5). Phytohormone applications significantly increased the biological yield, with the 100% ETc + MGA3 treatment achieving the highest yield in both seasons (Fig 5). This pattern was mirrored in the final grain yield, where the treatment efficacy followed a clear descending order: 100% ETc + MGA3 > 80% ETc + MGA3 > 100% ETc + MASA > 80% ETc + MASA > 60% ETc + MGA3 > 60% ETc + MASA > 100% ETc > 80% ETc > 60% ETc.

### Effect of phytohormone application on yield components and final grain yield

**Fig 6:**
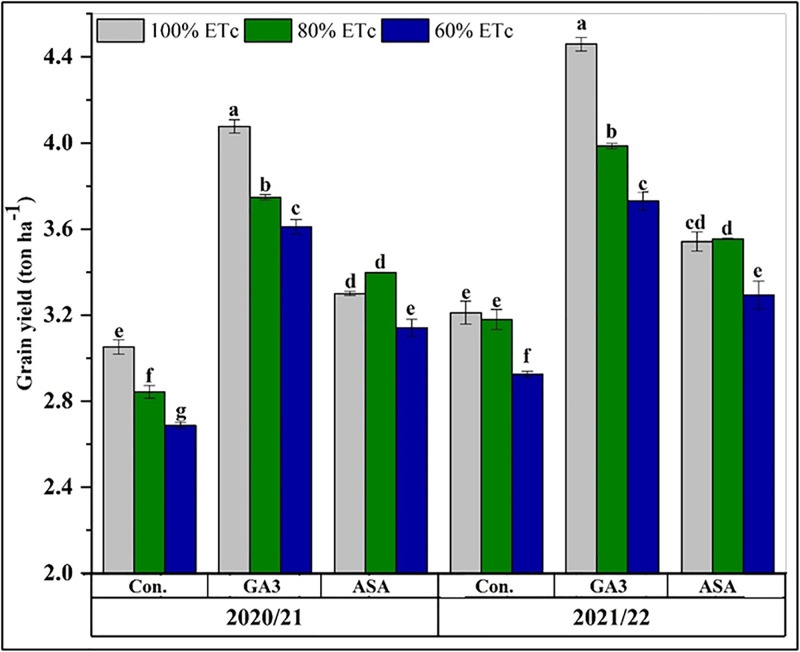
The effects of deficit irrigation and microbial phytohormones on wheat (Masr-1) grain yield during the 2020/2021 and 2021/2022 seasons. CK: control, GA3; microbial gibberellic acid (MGA3), ASA; microbial ascorbic acid (MASA). Data are presented as means, line bars indicate ± standard errors, and the different upper letters demonstrate significant differences at P < 0.05 level according to Tukey’s multiple range tests at P < 0.05.

Deficit irrigation significantly reduced grain yield, with the lowest yield occurring at 60% ETc in the second season (Fig 6). On average across both seasons, yield declined by 4% at 80% ETc and 10% at 60% ETc compared to full irrigation (100% ETc). The application of microbial phytohormones effectively mitigated this reduction. Treatments with MGA3 and MASA consistently yielded more than deficit irrigation alone, with MGA3 proving superior to MASA. The highest grain yield was achieved with the 100% ETc + MGA3 treatment, which increased the average yield by 36% compared to the 100% ETc control (CK). A consistent hierarchy of treatment efficacy was observed, descending as follows: 100% ETc + MGA3 > 80% ETc + MGA3 > 60% ETc + MGA3 > 80% ETc + MASA > 100% ETc + MASA > 60% ETc + MASA > 100% ETc > 80% ETc > 60% ETc. Overall, grain yields were higher in the first season across all treatments (Fig 6).

### Effects of deficit irrigation and microbial phytohormone treatments on water productivity

**Fig 7:**
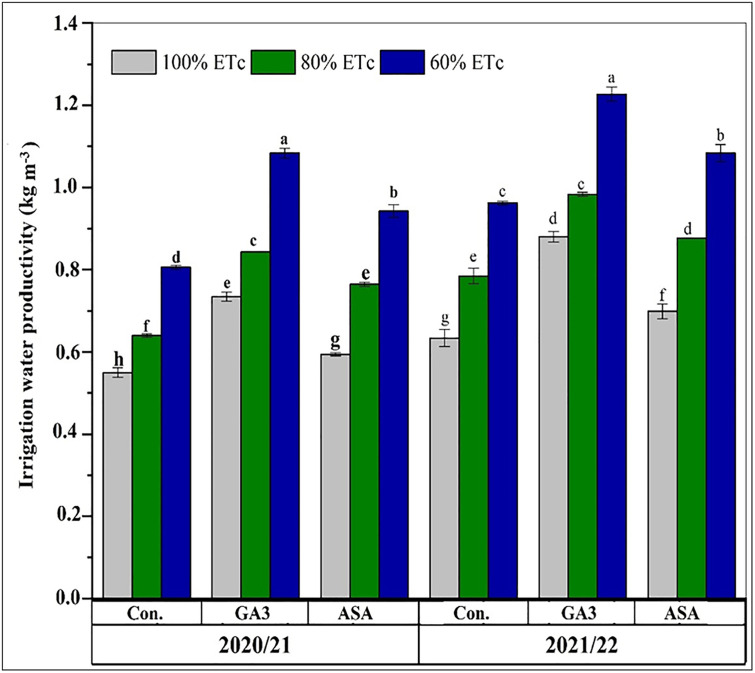
The effects of deficit irrigation and microbial phytohormones on wheat (Masr-1) irrigation water productivity (IWP) during the 2020/2021 and 2021/2022 seasons. CK: control, GA3; microbial gibberellic acid (MGA3), ASA; microbial ascorbic acid (MASA). Data are presented as means, line bars indicate ± standard errors, and the different upper letters demonstrate significant differences at P < 0.05 level according to Tukey’s multiple range tests at P < 0.05.

Deficit irrigation significantly improved IWP; compared to the 100% ETc treatment, IWP increased by 34% and 10% at 80% and 60% ETc in the first season, and by 39% and 10% in the second season, respectively (Fig 7). The application of microbial phytohormones further enhanced water use efficiency, with both MGA3 and MASA significantly increasing IWP and CWP compared to deficit irrigation alone (p < 0.05). The maximum IWP in both seasons was achieved with the 60% ETc + MGA3 treatment, which was significantly superior to the 60% ETc + MASA treatment at the same irrigation level (Fig 7).

**Fig 8:**
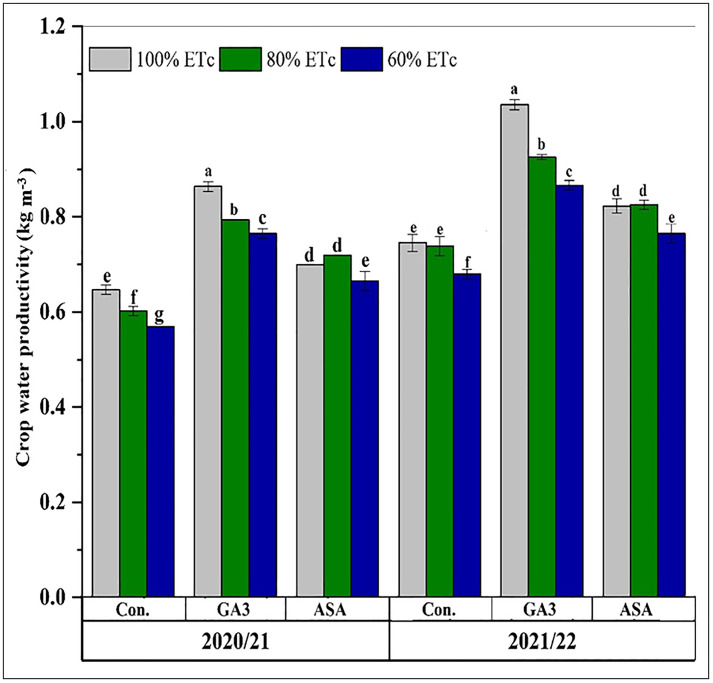
The effects of deficit irrigation and microbial phytohormones on wheat (Masr-1) crop water productivity (CWP) during the 2020/2021 and 2021/2022 seasons. CK: control, GA3; microbial gibberellic acid (MGA3), ASA; microbial ascorbic acid (MASA). Data are presented as means, line bars indicate ± standard errors, and the different upper letters demonstrate significant differences at P < 0.05 level according to Tukey’s multiple range tests at P < 0.05.

Analysis of CWP revealed that the 80% ETc + MGA3 treatment significantly enhanced it in both seasons compared to deficit irrigation with MASA or without phytohormones (P < 0.05, Fig 8). Conversely, the 60% ETc treatment without phytohormones resulted in the lowest CWP. A clear hierarchy of treatment efficacy was established for CWP, descending as follows: 100% ETc + MGA3 > 80% ETc + MGA3 > 60% ETc + MGA3 > 80% ETc + MASA > 100% ETc + MASA > 60% ETc + MASA > 100% ETc > 80% ETc > 60% ETc. The minimum IWP was recorded for the 100% ETc + MASA treatment during the 2021/2022 season. Across all treatments, the mean values for both CWP and IWP were higher in the second growing season compared to the first season.

### Redundancy analysis (RDA) of the efficacy of MGA3 on grain yield and crop water productivity (CWP)

**Fig 9:**
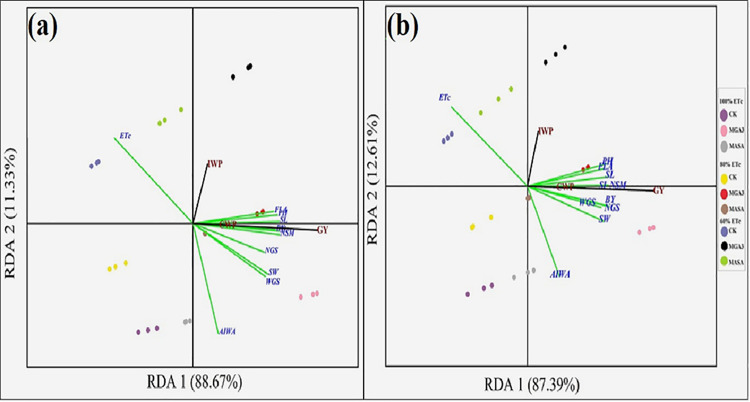
Redundancy analysis of the effects of crop evapotranspiration (ETc), actual irrigation water applied (AIWA), major plant growth, and yield attributes on irrigation water productivity (IWP), crop water productivity (CWP), and crop yield. The response variables are yield, CWP, and IWP. The explanatory variables are AIWA, MWD, plant height (PH), flag leaf area (FLA), spike length (SL), number of spikes per m2 (NSM), number of grains per spike (NGS), spike weight (SW), grain weight per spike (WGS), seed index (SI), biological yield (BY), and grain yield (GY). **a)** First season (2020/2021); **b)** Second season (2021/2022).

The Redundancy Analysis (RDA) clearly illustrated the relationships between treatments, plant traits, and water productivity for both growing seasons (Fig 9a and 9b). The results demonstrated a strong positive association between the MGA3 treatments (from 60% to 100% ETc) and enhanced grain yield and CWP, which were closely linked to improved plant height, flag leaf area, biological yield, spike number, seed index, and grain number per spike. In contrast, a negative relationship was observed where IWP decreased as the AIWA increased, a trend associated with the CK and MASA treatments.

The analysis confirmed significant correlations across both seasons: CWP and grain yield were negatively correlated with ETc, while plant height and biological yield showed a strong positive correlation with CWP. Biological yield and the number of spikes per m² were identified as the most influential traits for increasing CWP and grain yield. Conversely, AIWA and ETc were the primary restricting factors for IWP. The RDA conclusively showed that foliar MGA3 application was superior to both MASA and the CK in improving IWP and CWP, with crop water productivity being primarily governed by grain yield and seed index.

## Discussion

Our study demonstrates that deficit irrigation imposes significant physiological stress on wheat, leading to substantial reductions in growth and yield. Nevertheless, the foliar application of microbial phytohormones, particularly MGA3, effectively mitigates these adverse effects and enhances water productivity. These findings align with and extend the current understanding of plant stress physiology and sustainable water management in arid zones.

Beyond their hormonal activity, microbially produced phytohormones may confer additional benefits through the presence of microbial-associated molecular patterns (MAMPs) or co-produced metabolites that prime plant defense and growth pathways. This holistic bio-stimulant effect could explain the superior performance of MGA3 over pure chemical formulations reported in some studies, though further metabolomic profiling is warranted.

The highest crop ETc observed in our well-irrigated plots was approximately 472.1 mm, confirming that drip irrigation is more water-conserving than traditional flood methods. The inter-seasonal variation in ETc and AIWA can be largely attributed to higher average wind speed in the first season, which intensified atmospheric drought and crop water demand, a phenomenon well-documented in arid environments [38,56].

Improving IWP is an essential agronomic goal to improve crop production under drought-stress conditions. Increased deficit irrigation levels substantially enhanced IWP while greatly lowering CWP in both growing seasons. Our results on water productivity present a nuanced picture that corroborates and extends existing knowledge. The increase in IWP under deficit irrigation is a well-documented phenomenon, also reported by Saad et al., 2023 [15], where water savings led to higher efficiency per unit of water applied. However, the concurrent drop in CWP highlights the trade-off between water savings and total biological output. The significant innovation here is that the application of MGA3 and MASA improved both IWP and CWP under deficit conditions compared to the untreated CKs, effectively ameliorating this trade-off by supporting higher biomass and grain production per unit of water consumed (Table 3). This can be attributed to maintaining grain while reducing water consumption under deficit irrigation. This result aligns with Zhao et al., 2020 [25], who revealed that IWP values for winter wheat declined by 7.12% and 4.78% under moderate (40–50% field water capacity) and severe (30–40% field water capacity) water stress, respectively. CWP can be enhanced by either increasing production per unit of water utilized or decreasing water usage per unit of production yield. Elevated levels of deficit irrigation markedly inhibited plant growth, adversely affecting CWP [9,12]. The current study reveals that IWP and CWP improved with the application of 60% ETc combined with MGA3 or MASA for IWP, and 100% ETc with MGA3 for CWP in both seasons. These findings suggest that GA3 and ASA positively mitigate the adverse effects of water stress on wheat plants. MGA3 or MASA-treated wheat plants under deficit irrigation exhibit reduced water loss and enhanced leaf water potential and carbon uptake rates, resulting in higher CWP values compared to deficit irrigation alone [31,33,57]. Our results indicate that exogenously applied phytohormones effectively improved the conversion of water into grain under 80% and 60% of ETc, with 80% ETc yielding similar CWP to 60% ETc.

The observed decline in morphological and yield parameters under deficit irrigation is a classic stress response, resulting from impaired cell division, nutrient uptake, and photosynthetic efficiency [11,12]. Our findings align with previous research on wheat under arid conditions. For example, Saad et al., 2023 [15] observed a significant reduction in wheat grain yield and yield components when irrigation was reduced to 50% of ETc, confirming that severe deficit irrigation often comes at the cost of productivity. In our study, the 60% ETc treatment without phytohormones similarly resulted in a substantial yield decline, reinforcing this challenge. These improvements can be attributed to increased moisture availability, which enhances nutrient uptake in the root zone. Reddy et al., 2024 [58] observed that higher uptake of nutrients and sufficient water content stimulated meristem activity, resulting to higher wheat growth, grain production, and its components. Phytohormone treatments enhanced plant growth indicators, such as height and seed index, and yield in both growth seasons compared to deficit irrigation alone. Nevertheless, the increased growth and production of wheat in this study showed that the application of MGA3and MASA mitigated the negative impacts of the drought.

Our findings revealed that in both seasons, irrigation deficit significantly decreased plant height. Stress from a water deficit reduces soil moisture, increasing competitive for water. As a result, plants allocate more resources to roots growth, which can reduce shoot height [59]. Haque et al., 2022 [60] reported similar results, noting that water deficit decreased plant height. They pointed out that insufficient water availability adversely affects plant growth due to a decrease in cell expansion and cell division. Compared to non-foliar applications, MGA3and MASA resulted in higher average plant heights in both growing seasons; the effects of the two treatments differed significantly. Phytohormones like bio-gibberellins and bio-ascorbic stimulate cell elongation and division, resulting in taller plants [61,62]. Greater height enhances sunlight capture, boosts photosynthesis, and increases biomass accumulation. In this regard, Farad et al., 2025 [63] found that foliar GA3 treatment enhanced plant height and biomass in wheat under water deficit.

The flag leaf plays a crucial role in determining spike yield, particularly during grain filling. Deficit irrigation slightly reduced flag leaf area, of wheat plants, though the change was not significant. This is because the water deficit limited photosynthesis, hindering leaf development and preventing full extension. Also, Ahmad et al., 2018 [24] pointed out that one of the most important mechanisms in plants to mitigate water deficit stress is the reduction of leaf area. Flag leaf area was significantly increased by the application of MGA3 or MASA across all irrigation rates (100%, 80%, and 60% ETc) in both growing seasons. Phytohormone application regulates cell growth and division, thereby enhancing photosynthesis and promoting plant growth. Godha et al., 2020 [64] found that applying GA3 and SA at 150 and 200 ppm boosted the flag leaf area in wheat production plots.

Spike weight and length are traits strongly linked to drought susceptibility index in wheat. In the present study, spike length and weight were significantly reduced in both growth seasons under 80% ETc and 60% ETc. Previous studies indicate that stress can impede the growth of vegetation and reproductive organs [15,24]. As the flag leaf and spike develop simultaneously, stress at a site affects both organs simultaneously. The study found that applying MGA3 or MASA at irrigation rates of 100%, 80%, and 60% ETc effectively enhanced spike length and weight in both growing seasons. MGA3 and MASA can act as protective agents, alleviating the adverse effects of water deficit stress on wheat and enhancing performance under both stress and optimal conditions [32,63]. Spike length and weight rose in the second season compared to the first, regardless of treatment. High temperatures in March likely hindered spike growth more in the first season than in the second.

Deficit irrigation notably decreased the number of grains per spike and seed index in wheat plants, while having a minimal effect on spikes per square meter. Previous studies on wheat have shown that water stress negatively effects the number of grains per spike and grain weight per spike, among other traits [14,15,65]. Similarly, Yu et.al., 2020 [66] found that drought-stressed wheat (*Triticum aestivum* L.) had fewer spikes, tillers, grains per plant, and lower grain weight. The key contribution of our research is demonstrating that foliar phytohormones can effectively mitigate this yield penalty. While Saad et al., 2023 [15] documented the yield loss under 50% ETc, our results show that with the application of MGA3, wheat plants under 60% ETc achieved yields that were competitive with, and in some cases superior to, the untreated CK at 80% ETc. This suggests that microbial phytohormones can be a vital tool for implementing more aggressive water-saving strategies without incurring the severe productivity losses reported in earlier studies. MGA3 was superior to MASA in improving the number of spikes per m^2^, grain weight and number per spike, and weight of 1000-grain weight. The findings highlight the importance of applying bio-gibberellins (bio-GA3) or bio-ascorbic acid (MASA) to improve wheat growth indicators and mitigate the impacts of water stress on the plants.

Biological yield (ton ha^-1^) and grain yield (ton ha^-1^) were significantly reduced under both 80% ETc and 60% ETc due to reduced photosynthesis under water deficit, which adversely affected leaf growth and, consequently, crop productivity. However, at 80% ETc, the drop in biological yield was moderate, as mild drought did not severely impair photosynthetic capacity. Yu et al., 2020 [66] and Haque et al., 2022 [60] observed that water deficit stress reduced wheat yields. Similarly, Zhang et al., 2018 [67] found that water deficit caused a 27.5% drop in grain yield and a 25% loss in biomass. These outcomes closely match our recent findings.

Our results show that foliar sprays of MGA3 and MASA substantially increased wheat biological and grain yields under irrigation deficit stress. This improvement may be attributed to enhanced physiological processes that promote plant growth and development, resulting in higher yield and its components [31,63]. Phytohormones enhance cell division and elongation, resulting in larger, more uniform grain-bearing structures. Our findings on the beneficial effects of phytohormones on wheat production under water deficit stress are consistent with those of Wakchaure et al. [12], who found that bioregulators enhance crops nutrient and water use efficiency, resulting in higher yields. Similar findings were reported by Alotaibi et al., 2023 [13], who observed that growth regulators reduced the adverse effects of water deficit stress and increased wheat growth and productivity. Grain yields across all treatments were lower in 2020−21 than in the 2021−22 season, demonstrating that responses to phytohormones and deficit irrigation are influenced by inter-annual weather variability. The unusually high March temperatures in the 1^st^ season likely shortened grain-filling period, hindering plant development and resulting in lower yields despite initially promising growth [68].

The practical implications for farmers in arid regions are significant. Our results support an integrated strategy combining deficit irrigation with foliar microbial phytohormones. For instance, applying 80% ETc with MGA3 provides an optimal balance, offering significant water savings while maintaining high yield and CWP. Under extreme water scarcity, the 60% ETc + MGA3 strategy emerges as a viable contingency plan, maximizing IWP and providing a salvageable yield. The use of microbial sources for these phytohormones further enhances the sustainability of this approach, aligning with the growing demand for environmentally friendly biostimulants in agriculture.

The use of *Fusarium incarnatum* and *Saccharomyces cerevisiae* for phytohormone production not only provides a sustainable sourcing strategy but also opens avenues for integrating microbial fermentation into local agricultural input systems. Future work should explore the scalability, formulation, and field stability of these microbial products, as well as their effects on soil microbiota and long-term soil health in arid farming systems.

## Conclusions

This study demonstrates that foliar application of microbial phytohormones, particularly MGA3, is a highly effective strategy for mitigating the adverse effects of deficit irrigation on wheat grown in arid calcareous soils. Over two growing seasons, our results demonstrated clear and measurable outcomes. The application of MGA3 under full irrigation (100% ETc) achieved the highest grain yield, significantly outperforming both the untreated control (CK) and MASA.

Crucially, the integration of MGA3 with deficit irrigation regimes proved to be a viable water-saving strategy. Under a moderate water deficit (80% ETc), MGA3 application maintained a high grain yields comparable to fully irrigated plots without phytohormones, while simultaneously enhancing water productivity. In scenarios of severe water scarcity (60% ETc), MGA3 treatment was key to maximizing output per unit of water applied, achieving the highest IWP. Redundancy analysis confirmed that these improvements in yield and water-use efficiency were driven by MGA3’s positive effect on fundamental agronomic traits, including biological yield, plant height, and seed index.

To build upon on these findings, future research should prioritize several key areas. First, multi-location trials are essential to validate the efficacy and consistency of this approach across diverse agro-climatic conditions and soil types. Second, more in-depth physiological measurements are needed to elucidate the precise mechanisms of action, such as effects on stomatal behavior, antioxidant enzyme profiles, and hormonal crosstalk. Finally, an economic analysis is needed to assess its cost-benefit ratio and practical adoption potential for farmers.

In summary, our results show that MGA3 can safeguard wheat productivity and enhance water-use efficiency under both moderate and severe water deficits. This establishes MGA3 as a potent phytohormonal tool for sustaining wheat production amid increasing water scarcity in arid regions.

Furthermore, the microbial origin of the phytohormones used in this study underscores the potential for developing sustainable, biologically derived inputs for water-stressed agriculture. Scaling up microbial production and optimizing delivery systems could make such strategies accessible to smallholder farmers in arid regions, contributing to both climate resilience and ecological intensification.
